# The inhibition mechanism of *Wolbachia* on mosquito-borne RNA virus replication as seen through lipid metabolism regulation

**DOI:** 10.3389/fmicb.2026.1749424

**Published:** 2026-04-13

**Authors:** Zifu Wang, Xiang Cui, Qingyong Zhang, Lili Zou, Jun Wang

**Affiliations:** 1 Hubei Provincial Clinical Research Center for Precise Prevention and Treatment of Gastrointestinal Cancer in the Elderly, The Second People’s Hospital of China Three Gorges University, Yichang, Hubei, China; 2 Key Laboratory of Tumor Microenvironment and Immunotherapy and Yichang Key Laboratory of Infection and Inflammation, College of Basic Medical Sciences, China Three Gorges University, Yichang, Hubei, China; 3 Yichang Central People's Hospital, Yichang, Hubei, China

**Keywords:** lipid metabolism, mosquito-borne RNA viruses, replication complex, resource competition, *Wolbachia*

## Abstract

Mosquito-borne RNA viruses, including but not limited to the dengue virus (DENV), Zika virus (ZIKV), and chikungunya virus (CHIKV), pose serious threats to global public health. Current countermeasure approaches are frequently plagued by insufficient coverage, susceptibility to drug resistance, and poor sustainability. *Wolbachia*, a natural symbiont within mosquitoes, has been shown to block the replication and transmission of mosquito-borne RNA viruses. In recent years, increasing attention has focused on the mechanism of its antiviral action, which involves the regulation of host lipid metabolism. Here, we systematically reviewed the mechanisms by which mosquito-borne RNA viruses disrupt normal lipid metabolism in host cells, elucidating how these viruses rely on host lipids to achieve invasion and form replication complexes. Multiple pathways of *Wolbachia* disrupting lipid metabolism are highlighted, including rearranging the host lipid environment, competing with viruses for key metabolic resources, regulating mitochondrial-lipid droplet interactions, and altering membrane fluidity. The translational medicine and public health applications of *Wolbachia* strains were explored, holding potential for advancing novel antiviral strategies based on metabolic disruption.

## 1 Background

Over the past 5 years (2021–2025), monitoring data from the World Health Organization (WHO) indicated that mosquito-borne RNA viruses continue to pose a significant global public health burden (World Health Organization, 2025). Dengue virus (DENV) remains the most widespread, with more than 7.6 million reported cases and over 3,000 deaths globally by April 2024, particularly affecting Southeast Asia, Latin America, and parts of Africa (Dengue–Global Situation, 2024). Zika virus (ZIKV), although showing a declining epidemic trend, persists at low-level transmission in over 90 countries across Latin America, the Caribbean, Southeast Asia, and Africa (Zika Epidemiology, 2024; Zika Virus, 2022; Rabe et al., 2025). Chikungunya virus (CHIKV) has re-emerged in cyclical outbreaks, with expanding transmission reported in 119 countries by late 2024, placing billions of people at risk (Chikungunya Epidemiology, 2025; Outbreak of Chikungunya Virus Poses Global Risk, Warns WHO, 2025). Yellow fever virus (YFV) cases have increased in the Americas, with recent outbreaks exhibiting high case fatality rates (Yellow Fever–Region of the Americas, 2025), while West Nile virus (WNV) continues to show fluctuating but overall rising incidence in Europe and North America, alongside geographic expansion (West Nile Virus, 2017; West Nile Virus–Barbados, 2024). Japanese encephalitis virus (JEV) remains endemic in Asia, with sporadic emergence in new regions, underscoring the sustained and evolving threat posed by mosquito-borne RNA viruses worldwide (Japanese Encephalitis, 2024).

**Figure 1:**
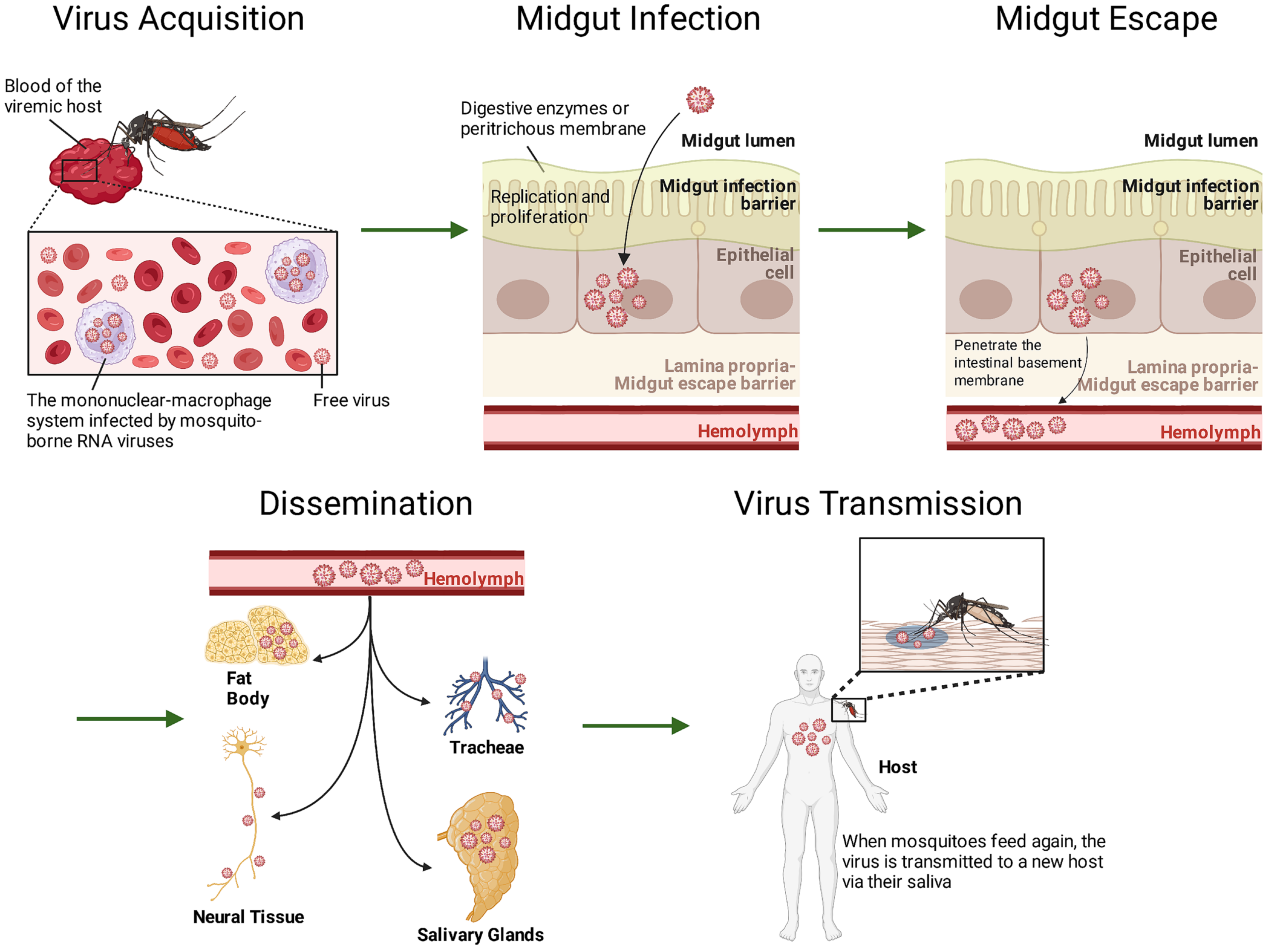
The infection and transmission process of mosquito-borne viruses within vector mosquitoes.

Mosquito-borne RNA viruses typically initiate infection within the midgut epithelial cells of mosquitoes (Jia et al., 2023; Zhu et al., 2021; Wu et al., 2025). After a female mosquito feeds on blood, the virus enters the midgut lumen with the blood meal (Wu et al., 2025). It must first breach the midgut infection barrier and replicate within the epithelial cells (Jia et al., 2023; Zhu et al., 2021; Wu et al., 2025). Subsequently, newly synthesized viral particles cross the basement membrane into the hemocoel and spread through the hemolymphatic system to secondary target tissues, particularly the salivary glands (Jia et al., 2023; Zhu et al., 2021; Wu et al., 2025). Extensive replication within salivary gland cells and subsequent release into saliva represent critical steps enabling viral transmission (Jia et al., 2023; Zhu et al., 2021; Wu et al., 2025) (Figure 1). The efficiency of viral replication within the mosquito and its transmission process from the midgut to the salivary glands are regulated by multiple factors, including the host’s genetic background, innate immune pathways, and symbiotic microorganisms (Jia et al., 2023; Zhu et al., 2021; Wu et al., 2025; Loterio et al., 2024). These factors collectively determine the mosquito’s transmission capacity.

Mosquito-borne RNA viruses are exhibiting characteristics of multicentric coexistence, climate-driven transmission, and geographic expansion (Carreto et al., 2022; Nelson and Ploss, 2024). The widespread and persistent prevalence of these RNA viruses has exposed the limitations of existing control systems, including insufficient vaccination coverage, lack of specific antiviral treatments, and increasing resistance of mosquito vectors to traditional control chemicals (Rakotoarimanana et al., 2025; Obi et al., 2021; Gan et al., 2021; Bowman et al., 2016). Among mosquito-borne RNA viruses, while vaccine breakthroughs have been achieved for some pathogens, such as the live attenuated 17D vaccine for YFV (World Health Organization, 2025; Litvoc et al., 2018), the first approved vaccine for CHIKV (World Health Organization, 2025; Weaver and Lecuit, 2015; Maurer et al., 2024; Liao et al., 2025; Ribeiro Dos Santos et al., 2025), and inactivated or attenuated vaccines for JEV (Campbell et al., 2011; Yun and Lee, 2014; Lopalco and Biasio, 2024). However, the quadrivalent dengue vaccine (e.g., Dengvaxia) remains significantly limited in its target population and protective efficacy (World Health Organization, 2025). Most other mosquito-borne RNA viruses still lack safe, effective, and widely applicable vaccines to date (World Health Organization, 2025; Plourde and Bloch, 2016; Chancey et al., 2015; Singh et al., 2025). Consequently, there is an urgent need to develop novel and sustainable strategies for preventing and controlling mosquito-borne RNA viruses. This paper focuses on *Wolbachia*, a Gram-negative bacterium. Building upon a review of its application progress, it emphasizes the analysis of its disease-resistant effects achieved through regulating host lipid metabolism, offering new insights into the interactions among bacteria, hosts, and pathogens.

*Wolbachia* is a type of endosymbiotic bacterium that is widely present in insects (Taha et al., 2025; Landmann, 2019). This species exhibits extremely high intraspecific diversity and is typically classified into multiple “supergroups” based on phylogenetic relationships (Wang et al., 2020). Among the currently identified *Wolbachia* supergroups, the A and B lineages are most prevalent, primarily residing within insects (Wang et al., 2020) (Table 1). The functional phenotype of *Wolbachia* exhibits host- and strain-dependent characteristics, manifesting as either an antiviral or non-antiviral state (Moreira et al., 2009; Fox et al., 2024). Specifically, A supergroup strains such as *w*Mel and *w*AlbB significantly suppress the replication and transmission of multiple mosquito-borne RNA viruses in *Aedes aegypti* (Moreira et al., 2009; Fox et al., 2024). Conversely, certain *Wolbachia* strains (e.g., *w*Pip or naturally infected symbiotic types) exhibit neutral effects on viral infection, indicating that their antiviral effects are highly strain- and host-dependent (Glaser and Meola, 2010; Micieli and Glaser, 2014). Although lacking stable antiviral activity, such strains can significantly influence host population structure by inducing cytoplasmic incompatibility (CI), thereby playing a crucial role in vector insect control (Sicard et al., 2021). Strains closely associated with mosquito-borne virus transmission disruption predominantly originate from the A supergroup (Wang et al., 2020; Gu et al., 2022; Chouin-Carneiro et al., 2020; Ant et al., 2018). The *w*Mel strain, first isolated from *Drosophila melanogaster*, has been successfully and stably introduced into mosquito species such as *Ae. aegypti* (Wang et al., 2020; Gu et al., 2022). It has become one of the most representative *Wolbachia* types in current research and applications for the biological control of mosquito-borne diseases (Wang et al., 2020; Gu et al., 2022).

**Table 1:** Major *Wolbachia* lineages used in mosquito-based disease control.

Strain	Supergroup	Original/ Native Host	Key Features/ Uses	Application
*w*Mel (Gu et al., 2022)	A	*Drosophila melanogaster* (fruit fly)	Strong virus blocking; induces CI; widely used in releases for dengue control (e.g., World Mosquito Program).	Stable in hot climates but can struggle at very high temperatures.
*w*AlbA (Chouin-Carneiro et al., 2020)	A	*Aedes albopictus* (Asian tiger mosquito)	Moderate virus blocking; often co-infects with *w*AlbB in native hosts.	Density can reverse when transferred to other species.
*w*AlbB (Al-Amin et al., 2025)	B	*Ae. albopictus*	High virus blocking; strong CI; trialed successfully in some areas.	Performs well in high-temperature environments. One of the important strains widely used in tropical regions.
*w*Au (Ant et al., 2018)	A	*Ae. albopictus* or related	Very efficient virus transmission blocking; stable superinfections possible.	Promising for tropical deployments.
*w*Pip (Lilja et al., 2024)	B	*Culex pipiens* (house mosquito)	Induces strong CI; variable subtypes in natural populations.	Common natural infection; not typically used in biocontrol.
*w*MelPop (Ritchie et al., 2015; Jiménez et al., 2021)	A	Variant of *w*Mel	Strong virus blocking but shortens host lifespan (pathogenic).	Less used now due to fitness costs. The most potent dengue-blocking strain known to date.
*w*Stir (Schultz et al., 2018)	Not assigned	*Drosophila simulans*	Established stable infection in mosquito cells and significantly inhibited the replication of multiple flaviviruses, demonstrating strong antiviral potential.	Mechanism research, candidate virus-blocking strains

CI, cytoplasmic incompatibility.

**Figure 2:**
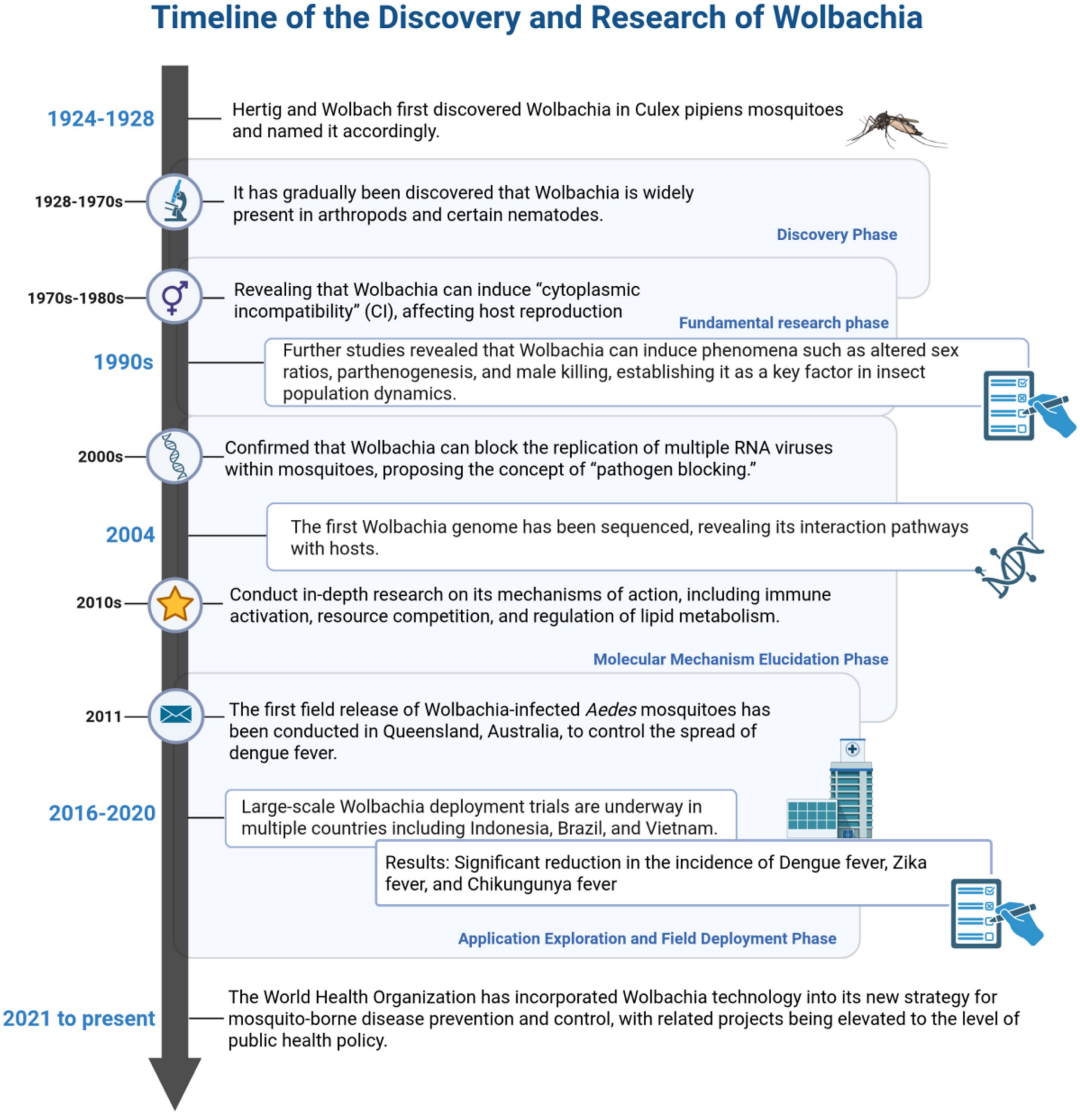
Timelines of discovery and research of *Wolbachia.*

Since *Wolbachia* can effectively block the replication and transmission of various mosquito-borne RNA viruses in mosquitoes, it has gradually become a research hotspot in the field of biological control of mosquito-borne diseases (Lu et al., 2020; Ekwudu et al., 2020). Since its discovery in the early 20th century, research on this bacterium has deepened due to its unique antiviral potential, gradually revealing its biological characteristics and host interaction mechanisms (Moreira et al., 2009; Hertig and Wolbach, 1924; Werren and Windsor, 2000; Yen and Barr, 1971; Sinkins et al., 1995; Wu et al., 2004; Ritchie et al., 2018; O'Neill et al., 2018; Utarini et al., 2021; Hoffmann et al., 2011). The initial discovery of the bacterium in mosquitoes dates back to 1924, when Hertig and Wolbach first identified it (Hertig and Wolbach, 1924). Subsequent milestones included the elucidation of mechanisms underlying reproductive isolation and population invasion in the late 20th century, as well as the successful artificial introduction of the bacterium into mosquito populations and the demonstration of its ability to block dengue virus transmission in the early 21st century, collectively establishing the theoretical and applied foundation for *Wolbachia*-based mosquito-borne disease control (Moreira et al., 2009; Hertig and Wolbach, 1924; Werren and Windsor, 2000; Yen and Barr, 1971; Sinkins et al., 1995; Wu et al., 2004; Ritchie et al., 2018; O'Neill et al., 2018; Utarini et al., 2021; Hoffmann et al., 2011) (Figure 2).

Based on these outstanding findings, *Wolbachia* has gradually transitioned from laboratory research to practical applications. As a naturally occurring symbiotic bacterium, it demonstrates significant potential in public health and translational medicine, with its ability to block viral transmission validated in multiple field trials (Taha et al., 2025; Landmann, 2019).

**Figure 3:**
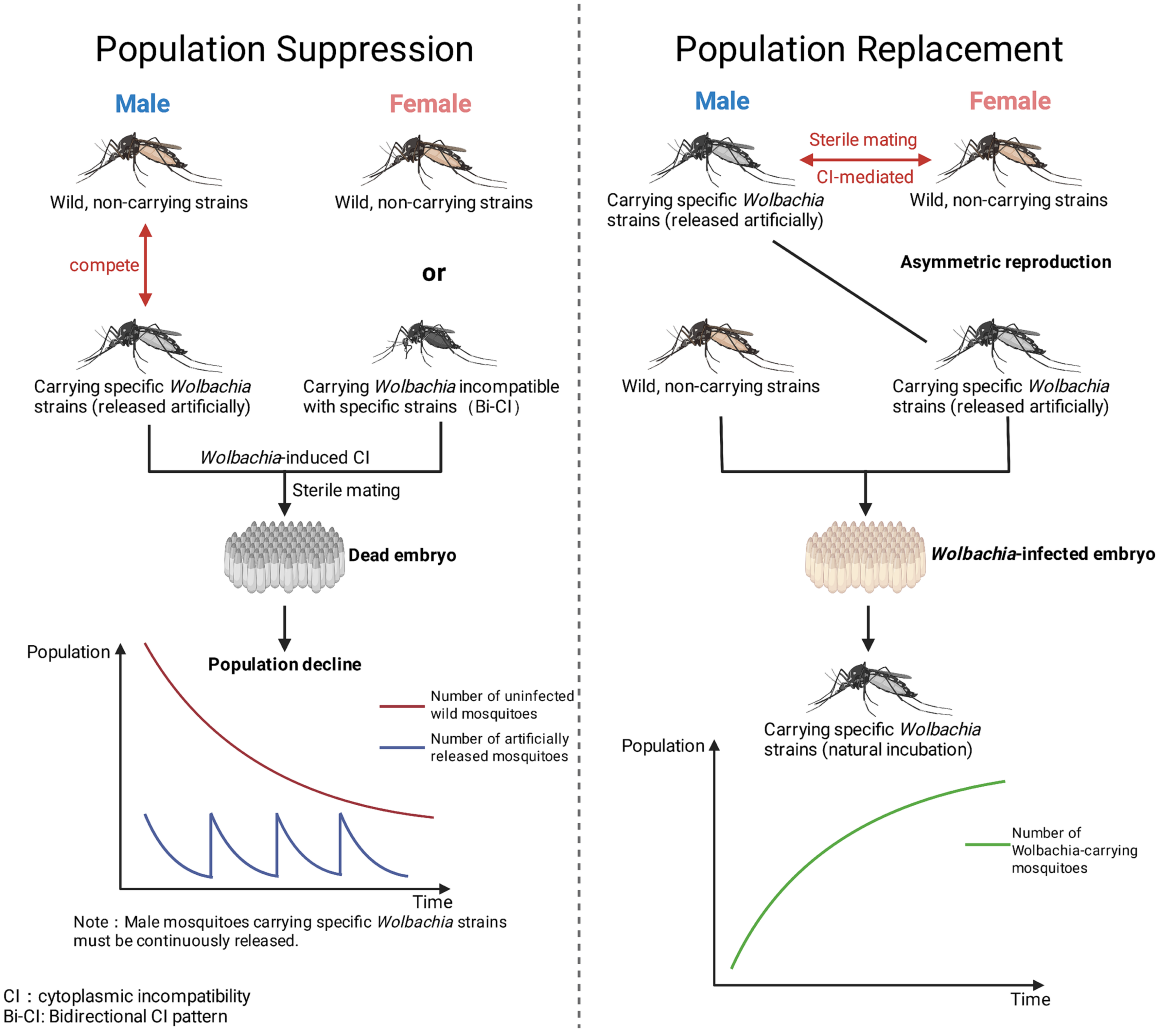
*Wolbachia*-based vector control strategies: population suppression and population replacement.

The primary applications of *Wolbachia* are population suppression and population replacement (Gu et al., 2022; Gaudillat et al., 2025) (Figure 3). The former primarily relies on *Wolbachia*-induced CI (Gaudillat et al., 2025; Moretti et al., 2018; Calvitti et al., 2012). By continuously releasing male mosquitoes carrying specific *Wolbachia* strains, these males mate with wild-type females to produce infertile eggs (Gaudillat et al., 2025; Moretti et al., 2018; Calvitti et al., 2012). This gradually reduces the density of target mosquito populations without introducing foreign genes (Gaudillat et al., 2025; Moretti et al., 2018; Calvitti et al., 2012). This strategy typically involves the release of male-only infected mosquitoes, emphasizing rapid population suppression and reversibility (Gaudillat et al., 2025; Moretti et al., 2018; Calvitti et al., 2012) (Table 2).

**Table 2:** Application of *Wolbachia* for population suppression in mosquito-borne disease control.

Region	Year	Experimental Methods	Results
Polynesia, French (O'Connor et al., 2012)	2012	Release of *w*Riversi-infected *Ae. aegypti* males, *Ae. Polynesiensis* (CP line).	Significant reduction in designated adult females mosquitoes.
Lexington, USA (Calvitti et al., 2012; Mains et al., 2016)	2012–2016	Release of *w*Pip-infected *Ae. aegypti* males (AR*w*P_US_ line).	Significant reduction in egg hatch rate, significant reduction in adult females.
Guangzhou, China (Zheng et al., 2019)	2019	Release of triple-infected (*w*AlbA + *w*AlbB + *w*Pip, HC line) *Ae. albopictus* males, and used in an open-release field trial of the combined IIT–SIT approach.	Combined IIT–SIT nearly eliminated two field populations of *Ae. albopictus* over a two-year period, without their replacement by released HC mosquitoes.
Plaeng Yao District, Thailand (Kittayapong et al., 2019)	2019	Release of *w*AlbA + *w*AlbB (ThAB line) infected *Ae. aegypti* males, combined with SIT, pilot trial.	84% reduction in egg hatch rate, 97% reduction in adult females.
Miami, USA (Mains et al., 2019)	2019	Release of *w*AlbB (WB1 line) infected *Ae. aegypti* males, large-scale trial.	Significant reduction in egg hatch rate, 78% reduction in adult females.
Fresno, USA (Crawford et al., 2020)	2020	Release of *w*AlbB (WB1 line) infected *Ae. aegypti* males, large-scale trial.	95% reduction in wild population.
Rome, Italy (Caputo et al., 2023; Caputo et al., 2020)	2020–2023	Release of *w*Pip (AR*w*P line) infected *Ae. albopictus* males, release ratio 0.7–1.1:1, pilot trial.	15–35% reduction in egg hatch rate (depending on year).
Innisfail, Australia (Beebe et al., 2021)	2021	Release of *w*AlbB (*w*AlbB2-F4 line) infected *Ae. aegypti* males, release ratio 5–10:1, large-scale trial.	Ignificant reduction in larval productivity, >80% reduction in adult population.
Merida, Mexico (Martín-Park et al., 2022)	2022	Release of *w*AlbB (WB2 line derivative) infected *Ae. aegypti* males, combined with SIT and IVM, release ratio 10:1, large-scale trial.	76–88% reduction in egg hatch rate, 55–75% reduction in biting females.
Houston, USA (Lozano et al., 2022)	2022	Release of *w*AlbB (WB1 line) infected *Ae. aegypti* males, large-scale trial.	93% reduction in *Ae. aegypti*, increase in *Ae. albopictus.*
Changsha, China (Zeng et al., 2022)	2022	Release of triple-infected (*w*AlbA + *w*AlbB + *w*Pip, HC line) *Ae. albopictus* males with IIT, large-scale trial.	97% reduction in egg hatch rate, 94% reduction in biting rate.
Yishun, Tampines, Bukit Batok, Choa Chu Kang, Singapore (Bansal et al., 2024; Lim et al., 2024b; Lim et al., 2024a)	2024	Release of *w*AlbB-infected *Ae. aegypti* males, IIT and IIT/SIT combination, operational program.	>90% reduction in wild population, 56–77% reduction in dengue incidence.
Ponce, Puerto Rico (Sánchez-González et al., 2025)	2025	Release of *w*AlbB infected *Ae. aegypti* males, large-scale trial.	49% reduction in wild population.
Virgin Gorda, British Virgin Islands (Ohm et al., 2025)	2025	Release of *w*AlbB (*Wb+*) infected *Ae. aegypti* males, large-scale trial.	No direct assessment of wild mosquito population density or suppression efficacy was conducted. Transported *Wolbachia*-infected male mosquitoes exhibited an 80–90% survival rate, a 3–5 day survival period in the field, a dispersal distance of 60–100 meters, and a recapture rate of 1–5%.

In contrast, the population replacement strategy aims to leverage *Wolbachia*’s maternal inheritance advantage within host populations, enabling stable expansion and long-term maintenance of *Wolbachia*-carrying mosquitoes in wild populations (O'Neill et al., 2018; Frentiu et al., 2014; Aliota et al., 2016; Ryan et al., 2019). This process typically involves releasing mosquitoes carrying *w*Mel or *w*AlbB strains to replace wild populations through CI, the transmission of mosquito-borne virus can be effectively interrupted (O'Neill et al., 2018; Frentiu et al., 2014; Aliota et al., 2016; Ryan et al., 2019). This resulted in the mosquito population exhibiting a phenotype characterized by significantly reduced or even lost transmission capacity for DENV, ZIKV, and CHIKV (O'Neill et al., 2018; Frentiu et al., 2014; Aliota et al., 2016; Ryan et al., 2019). Unlike population suppression, population replacement does not primarily target reducing mosquito numbers but instead diminishes their vector capacity by altering population composition (O'Neill et al., 2018; Frentiu et al., 2014; Aliota et al., 2016; Ryan et al., 2019) (Table 3).

**Table 3:** Application of *Wolbachia* for population replacement in mosquito-borne disease control.

Region	Year	Experimental Methods	Results
Cairns, Australia (Frentiu et al., 2014)	2014	Comparing DENV infection rates and transmissibility between *Ae. aegypti* carrying *w*Mel and control populations.	In wild-collected mosquito populations infected with *w*Mel virus, viral RNA copy numbers were 10^3^–10^4^ times lower than in the control group, and the DENV-2 infection rate decreased by 90%.
Townsville, Australia (O'Neill et al., 2018)	2018	Gradually release *Wolbachia*-infected *Ae. aegypti* throughout the city.	Achieving >90% infection coverage, dengue fever cases have virtually disappeared across the city, with effects maintained over the long term.
Queensland, Australia (Ryan et al., 2019)	2019	Release *Ae. aegypti* carrying *w*Mel *Wolbachia* into natural populations to establish stable populations in the wild.	*Wolbachia* achieved sustained high-level stable colonization in local mosquito populations for over 8 years, while reducing local dengue incidence by 95%.
Indonesia, Yogyakarta (Utarini et al., 2021; Indriani et al., 2020)	2020–2021	Randomized controlled trial (RCT) releases *Ae. aegypti* carrying the *w*Mel *Wolbachia.*	The deployment of *w*Mel *Wolbachia* reduced dengue incidence by about 77% compared with non-intervention areas; Subsequent analyses showed hospitalizations for severe dengue dropped by 76%, and overall cases by 73%. These reductions persisted for approximately 30 months post-intervention.
Rio de Janeiro, Brazil (Moledo Gesto et al., 2021)	2021	Release *w*Mel-infected *Ae. aegypti* (*w*MelRio) strains matched to the local genetic background (by vehicle and backpack), combined with continuous mosquito trapping and molecular detection to monitor *Wolbachia* establishment.	*Wolbachia* infection rates stabilized at approximately 50–70% (locally >80%) in the RJ1 area and at approximately 30–60% in the RJ2 area; infection rates in vehicle-release areas increased faster and reached higher steady-state levels than in backpack-release areas; the frequency of kdr resistance alleles in *Wolbachia*-positive *Ae. aegypti* populations matched and remained stable at levels consistent with local wild populations.
Niteroi, Brazil (Pinto et al., 2021)	2021	Releasing *Ae. aegypti* carrying *w*Mel in urban environments.	The incidence of dengue fever decreased by approximately 69% compared to the control area; The cases of Zika and chikungunya fever also dropped by 37 and 56%, respectively.
Bello, Medellín, and Itagüí, Colombian (Velez et al., 2023)	2023	The *w*Mel-infected *Aedes aegypti* mosquitoes were released in phases across the entire city. The intervention effectiveness was evaluated through interrupted time series analysis and negative-control case–control analysis.	When the Wolbachia establishment rate reached ≥60%, the incidence of dengue fever decreased by 95–97%. Individual-level analysis indicated that the probability of infection in the intervention areas decreased by approximately 45–47%.
Yishun and 14 other communities, Singapore (Lim et al., 2024b)	2024	Release *Ae. aegypti* carrying *w*Mel *Wolbachia* and assess the correlation between *Wolbachia* infection rates in mosquitoes.	Approximately 6–9 months after release, *w*Mel *Wolbachia* infection rates exceeded 80% in wild mosquito populations, and dengue cases in intervention areas decreased by approximately 77%.
Selangor, Malaysia (Hoffmann et al., 2024)	2024	Release *Ae. aegypti* carrying *w*AlbB into target communities; collect dengue incidence data through routine disease reporting systems during the monitoring period.	Compared to the control area, the overall average incidence of dengue fever decreased by 62.4%. In the release area, the average *w*AlbB infection frequency reached 82.3%. Model estimates indicate that achieving the assumed 100% *w*AlbB infection frequency could reduce dengue fever incidence by 75.8%.
Nakasi, Fiji (Lin et al., 2024)	2024	Two field trials were conducted using unmanned aerial vehicles to release *w*AlbB-infected *Ae. aegypti*, covering an area of approximately 2 square kilometers.	*Wolbachia* infection rates were successfully established and maintained at stable levels within the target mosquito population, with approximately 58.6% of *Ae. aegypti* detected as *Wolbachia* carriers during the final monitoring period.
Indonesia, Yogyakarta (Lim et al., 2025)	2025	Based on urban dengue fever surveillance data, an emulated target trial analysis was employed.	In areas adjacent to those not directly released *Wolbachia*-infected mosquitoes, dengue incidence still decreased by 45% and continued to decline over time.

By synergistically employing population suppression and population replacement strategies, we can not only safely and sustainably reduce the number of biting female mosquitoes but also effectively block the transmission of mosquito-borne viruses within the remaining mosquito populations. These results provide strong evidence for the practical application of *Wolbachia*. *Wolbachia* is an obligate intracellular symbiont that co-localizes with various mosquito-borne RNA viruses within the host cytoplasm (Bhattacharya et al., 2020; Rainey et al., 2016). The *Wolbachia*-associated blocking effect manifests during the early stages of viral entry and release of genomic RNA, primarily by inhibiting viral translation and replication (Bhattacharya et al., 2020; Rainey et al., 2016). *Wolbachia* induces the expression of RNA degradation-related factors (such as *RNase HI* (Hussain et al., 2023)) in host cells, thereby promoting the degradation of viral RNA genomes (Hussain et al., 2023). Although direct evidence regarding specific blocking sites of *Wolbachia* in different tissues remains limited, studies have demonstrated its high colocalization with RNA viruses in key mosquito tissues such as the midgut and salivary glands (Bhattacharya et al., 2020; van den Hurk et al., 2012; Amuzu and McGraw, 2016; Mejia et al., 2022). Given their shared utilization of host metabolic and replication resources within cells, this spatial overlap likely leads to competition for critical host factors, thereby limiting efficient viral replication and transmission (Bhattacharya et al., 2020; van den Hurk et al., 2012; Amuzu and McGraw, 2016; Mejia et al., 2022). The antiviral effects of *Wolbachia* are not mediated by a single mechanism but likely involve multiple processes, including activation of host immune responses, competitive occupation of viral replication-associated cellular sites, and alteration of the intracellular environment, *etc.* (Loterio et al., 2024; Mushtaq et al., 2024; Pan et al., 2018; Rainey et al., 2023). (Table 4). Among these, the mechanism of inhibiting viral replication by interfering with host lipid metabolism has garnered significant attention in recent years and is considered one of the primary contributors to the antiviral effects mediated by *Wolbachia* (Loterio et al., 2024; Mushtaq et al., 2024; Pan et al., 2018). This paper focuses on elucidating the role of lipid metabolism as a primary contributing pathway, though it does not exclude other mechanisms. It is known that lipid metabolism in host cells plays a crucial role in the life cycle of mosquito-borne RNA viruses, influencing viral invasion, envelope formation, and the construction of replication complexes (RC) (Oyarzún-Arrau et al., 2020; Martín-Acebes et al., 2011; Heaton and Randall, 2011; Koh et al., 2020; Heaton et al., 2010). An in-depth analysis of its molecular mechanism helps reveal the complex interactions among symbiotic bacteria, hosts, and viruses, while providing a theoretical basis and practical direction for the design and application of new intervention strategies against mosquito-borne RNA viruses.

**Table 4:** *Wolbachia*-mediated host responses and their constraints on the life cycle of mosquito-borne RNA viruses.

Suppressive effects	Research strains	Key host process or molecule	Site of occurrence	Mechanism	Affected viruses
Reduced initial viral infection efficiency (Lu et al., 2020)	*w*AlbB	Membrane fluidity, endocytic vesicle formation	Midgut epithelium	*Wolbachia* alters the membrane properties and endocytosis dynamics of midgut epithelial cells, reducing the probability of viral entry into host cells.	DENV, ZIKV
The formation or maintenance of viral replication factories is restricted (Loterio et al., 2024; Geoghegan et al., 2017; Hussain et al., 2013)	*w*Mel, *w*MelPop, *w*AlbB, *w*Pip	ER membrane rearrangement, membrane bending capability	ER	*Wolbachia* remodels the ER membrane structure, disrupting the formation and stability of virus-induced replication vesicles or DMVs.	DENV, WNV
Decreased Stability of Clone Factory Membranes (Geoghegan et al., 2017; Reyes et al., 2021)	*w*MelPop	Cholesterol, sphingolipids, phospholipids	ER	Reduces the availability of cholesterol, sphingolipids, and certain phospholipids, thereby compromising the membrane integrity of viral replication structures.	DENV
Educe the stability and replication efficiency of viral RNA (lack of direct evidence), degradation of RNA (Hussain et al., 2023; Koh et al., 2020; Geoghegan et al., 2017; Raquin et al., 2015; Asad et al., 2018; Zhang et al., 2013; Bhattacharya et al., 2022)	*w*MelPop, *w*Mel, *w*AlbB	Fatty acid β-oxidation, mitochondrial function, host factors associated with RNA modifications and translational homeostasis	Mitochondria, ER, Cytoplasm	Inhibits fatty acid β-oxidation and reduces energy metabolic flux, thereby limiting the energy-intensive viral RNA synthesis process (Koh et al., 2020; Geoghegan et al., 2017).	DENV, CHIKV (Raquin et al., 2015)
				Downregulate multiple host factors associated with RNA modification and translational homeostasis, such as AaDnmt2, pelo and DNMT2 *etc*., through host microRNA or direct transcriptional regulation (Asad et al., 2018; Zhang et al., 2013; Bhattacharya et al., 2022).	
				Induce cells to produce *RNase HI*, which degrades the viral RNA genome (Hussain et al., 2023).	
Reduced midgut escape efficiency (Terradas et al., 2017; Pujhari et al., 2023; Rancès et al., 2012; Jupatanakul et al., 2017; She et al., 2024)	*w*Mel, *w*AlbB, *w*MelPop	Antimicrobial peptides, immunomodulatory factors	Fat body, hemolymph	Activation of innate immune pathways (such as Toll, IMD, JAK–STAT, and RNAi) imposes certain limitations on the efficiency of viral transmission within the body cavity. However, most experiments demonstrate that upregulating immune effector genes is not a prerequisite for interfering with dengue virus replication.	DENV, O’nyong nyong virus (ONNV)
Limited utilization of host resources (She et al., 2024)	*w*AlbB, *w*MelPop	miRNA Network	Whole-cell	Regulate miRNA and host gene expression profiles to weaken viral hijacking of host transcriptional and translational resources.	DENV

The mechanism by which *Wolbachia* suppresses YFV remains unclear. While infection and transmission rates are reduced (Rocha et al., 2019), there is a lack of definitive molecular or cellular-level evidence supporting this effect. No studies have demonstrated *Wolbachia* suppression of JEV, and the underlying mechanism for such suppression also remains unknown. The two viruses mentioned above are not listed in the table. ER, endoplasmic reticulum.

## 2 The molecular basis of virus infection dependent on host lipids

The replication and transmission of mosquito-borne RNA viruses in the host are highly dependent on the participation and regulation of lipids. Lipids are the key components supporting the host cell membrane structure, while being deeply involved in the formation of RC, viral envelopes, and in virion assembly and release during mosquito-borne RNA virus infection. A variety of mosquito-borne RNA viruses have been demonstrated to actively reprogram the lipid metabolism of their to support their life cycle. Understanding lipid metabolism in the formation of host cell membrane systems and the virus’s remodeling of the host membrane environment to construct the RC provides the theoretical foundation for revealing how *Wolbachia*-induced alterations in host lipid metabolism can result in impaired viral replication.

### 2.1 Physiological processes of lipid metabolism in mosquito cells

**Figure 4:**
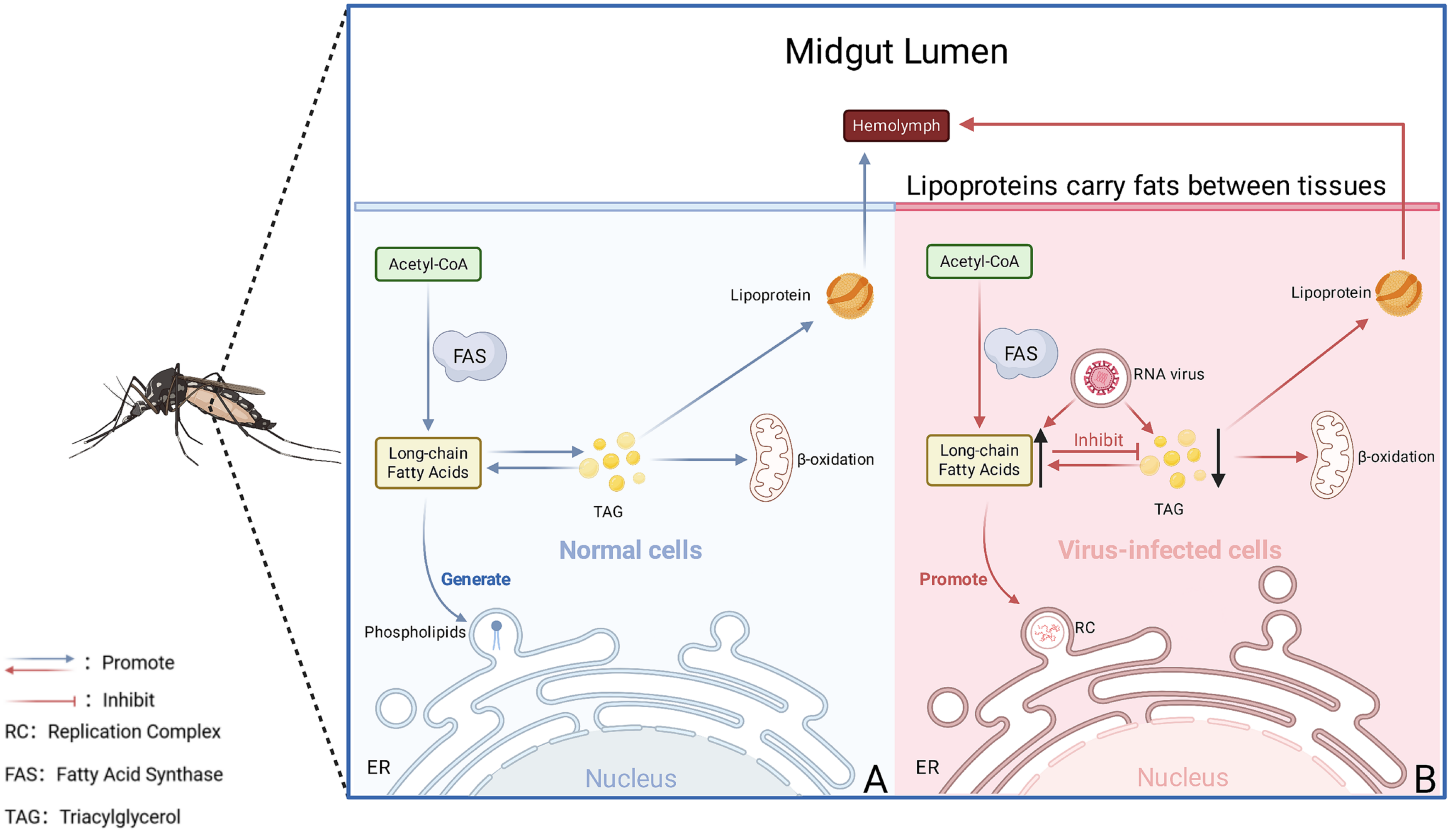
Comparison of lipid metabolism between normal cells and virus-infected cells. **(A)** Lipid metabolism profile of normal mosquito cells; **(B)** cellular metabolism profile following infection with mosquito-borne RNA viruses.

Under normal physiological conditions, lipid metabolism in mosquito cells maintains a dynamic balance of synthesis, storage, and utilization. Acetyl-CoA generates long-chain fatty acids under the catalysis of Fatty Acid Synthase (FAS); these fatty acids enter the phospholipid biosynthesis pathway within the endoplasmic reticulum (ER) for use in constructing membrane system (Chotiwan et al., 2022). The excess neutral lipids are stored in lipid droplets in the form of triacylglycerol (TAG) and are rapidly mobilized when the demand for energy or membrane raw materials increases (Barletta et al., 2016; Dos Santos et al., 2024); lipoproteins are responsible for transporting lipids between tissues (Gondim and Majerowicz, 2024); cholesterol and sphingolipids ensure the stability of membranes and signal transduction (Gold et al., 2020; Li et al., 2024; Fu et al., 2011) (Figure 4A); other lipid substances such as diacylglycerol (DAG), phosphatidylinositol (PI), and their phosphorylated derivatives (e.g., PIP_2_, PIP_3_) may participate in signaling pathways like PI3K/AKT and PKC, regulating metabolic and immune-related responses (Toprak, 2020; Pennington and Wells, 2002; Liu et al., 2015; Walkowiak-Nowicka et al., 2021). These molecular events are ultimately regulated by the body’s overall hormonal system, with the entire process achieving a delicate balance under the control of signals such as Juvenile Hormone (JH) and ecdysone (Liu et al., 2015; Dou et al., 2023; Wang et al., 2017). This highly flexible metabolic network enables mosquito cells to efficiently allocate lipids during physiological processes such as development and reproduction; it also creates conditions for viruses to reprogram these pathways and establish membrane systems suitable for replication after infection (Chotiwan et al., 2018).

### 2.2 The role of the host membrane system in RNA virus replication complex formation

**Figure 5:**
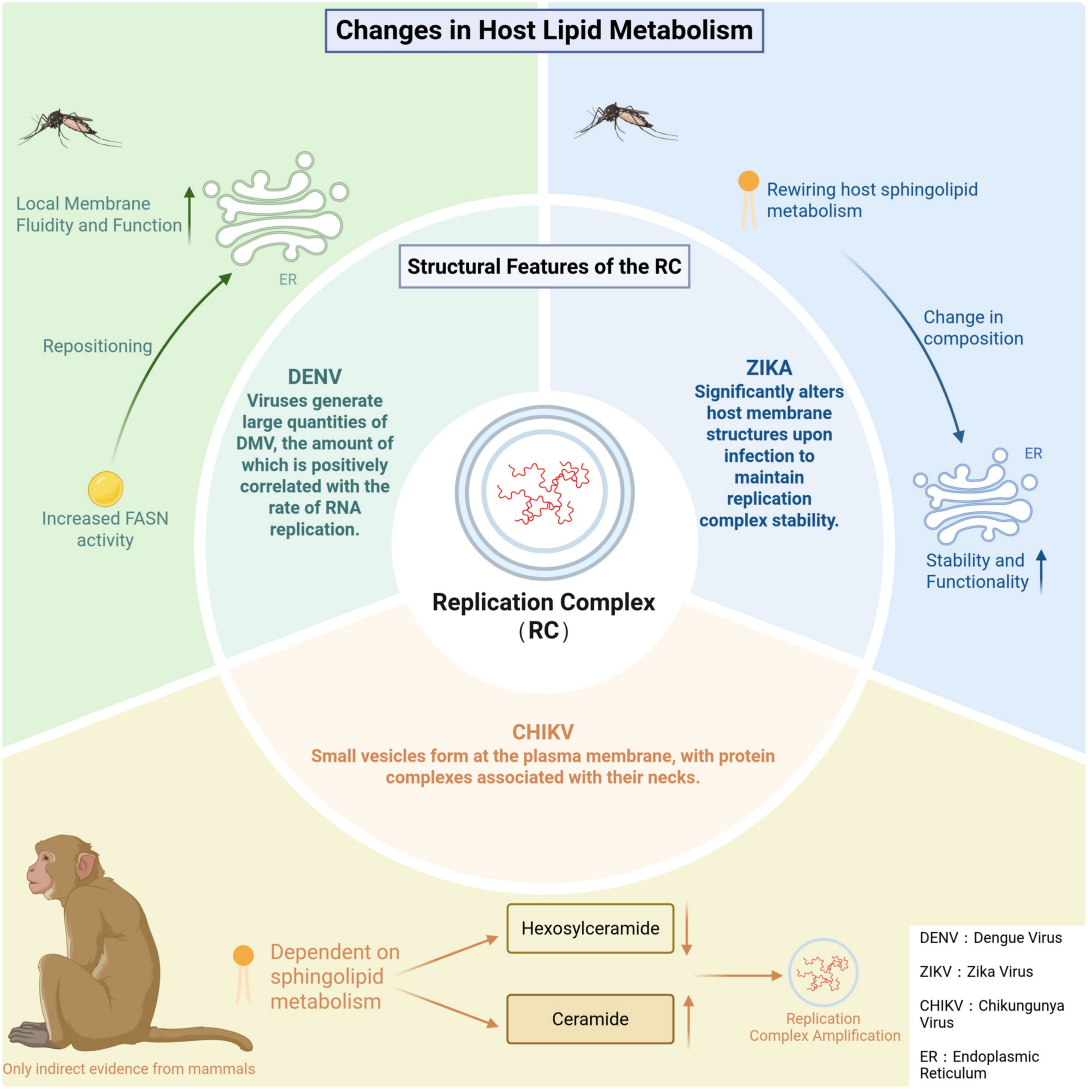
Comparative mechanisms of mosquito-borne RNA virus replication complex formation. For DENV and ZIKV, mechanisms are supported by experimental evidence from mosquito hosts. For CHIKV, the vertebrate host depicted indicates that current mechanistic evidence is primarily derived from vertebrate systems, highlighting the limited availability of mosquito-based data.

The host cellular membranes are not an isolated organelle, but a complete membranous network that can be modified and utilized by the virus after it infects the host (Diaz et al., 2010; Diaz and Ahlquist, 2012; Gao et al., 2025). In this context, RNA viruses assemble viral RC through these membrane structures (Miller and Krijnse-Locker, 2008; Paul and Bartenschlager, 2013; Romero-Brey and Bartenschlager, 2014; den Boon and Ahlquist, 2010; Zhang et al., 2020). This multimolecular complex comprises the viral RNA-dependent RNA Polymerase (RdRp), associated non-structural proteins, and multiple types of host factors, thereby driving viral genome replication and transcription (Miller and Krijnse-Locker, 2008; Paul and Bartenschlager, 2013; Romero-Brey and Bartenschlager, 2014; den Boon and Ahlquist, 2010; Zhang et al., 2020). In the early stages of infection, the virus induces significant morphological and functional remodeling of the host membrane system (Loterio et al., 2024; Welsch et al., 2009; Cortese et al., 2017; Vial et al., 2020; Neufeldt and Cortese, 2022). Subsequently, it reprograms host lipid metabolism to provide the necessary materials and energy (such as sphingomyelin (Leier et al., 2020)) for membrane structure formation (Loterio et al., 2024; Welsch et al., 2009; Cortese et al., 2017; Vial et al., 2020; Neufeldt and Cortese, 2022). Finally, the assembly and activation of the RC occur on the surface or within the lumen of these remodeled membrane structures (Loterio et al., 2024; Welsch et al., 2009; Cortese et al., 2017; Vial et al., 2020; Neufeldt and Cortese, 2022). To provide structural support and protect viral RNA from host recognition, most mosquito-borne RNA viruses trigger the remodeling of the host cell membrane system (Miller and Krijnse-Locker, 2008; Paul and Bartenschlager, 2013; Welsch et al., 2009; Lin et al., 2019; Romero-Brey and Bartenschlager, 2016). This process leads to the formation of specialized membrane structures, such as double-membrane vesicles (DMVs), spherules, or vesicle packets, which create a suitable environment for efficient viral replication (Miller and Krijnse-Locker, 2008; Paul and Bartenschlager, 2013; Welsch et al., 2009; Lin et al., 2019; Romero-Brey and Bartenschlager, 2016). This process relies on key enzymes involved in phospholipid synthesis, such as the host fatty acid synthase (FASN) and Lysophosphatidylcholine Acyltransferase (LPCAT), as well as proteins regulating membrane stability, such as sphingomyelin synthase (Hitakarun et al., 2022; Sornprasert et al., 2025; Hishikawa et al., 2014; Orchard et al., 2018) (Figure 4B). The mechanism has been confirmed in mosquito cells infected with various mosquito-borne RNA viruses, and the following section will specifically elaborate on the role of the host membrane system and lipid metabolism in the formation of replication factories, combining the research progress of different viruses. The ultrastructural architecture of DENV-infected mosquito cells (C6/36 cells, a mosquito cell line) utilizing transmission electron microscopy combined with three-dimensional reconstruction techniques (Junjhon et al., 2014). The results confirmed that the virus induces the formation of a large number of DMVs, and the quantity of DMVs is significantly correlated with the RNA replication rate (Junjhon et al., 2014). The activity of the host FASN is enhanced, and it is relocated to the replication factory region (Chotiwan et al., 2018). This change coincides with the timing of peak viral replication in *Ae. aegypti* and is involved in improving membrane fluidity and functional applicability (Chotiwan et al., 2018). Both ZIKV and DENV remodel endoplasmic reticulum membrane structures to form replication factories characterized by DMVs (Morita and Suzuki, 2021). However, multiple lipidomics studies indicate significant differences in the viruses’ dependence on host membrane lipid composition and metabolic pathways during replication (Heaton et al., 2010; Cortese et al., 2017; Leier et al., 2020; Mohd Ropidi et al., 2020; Tongluan et al., 2017). ZIKV infection more strongly disrupts sphingolipid and cholesterol metabolism, relying on these lipids to maintain the stability and function of the replication factory (Cortese et al., 2017; Leier et al., 2020; Mohd Ropidi et al., 2020). In contrast, DENV replication is more dependent on the phospholipid composition of the host endoplasmic reticulum membrane and fatty acid synthesis pathways (Heaton et al., 2010; Tongluan et al., 2017). This difference in dependence on the mosquito vector host membrane chemical environment may explain the inconsistent suppression effects observed for different viruses under *Wolbachia* infection conditions. To date, there remains a lack of direct evidence regarding the mechanism by which CHIKV forms RC in mosquito hosts. However, CHIKV infection relies on host sphingolipid metabolism and reshapes this metabolic process *in vitro* cell cultures, especially by reducing Hexosylceramide (HexCer) and increasing ceramide levels to support the formation of viral RC and the expansion of infection (Noble et al., 2025; Tan et al., 2022; Laurent et al., 2022) (Figure 5). In conclusion, the remodeling of the host membrane system and the regulation of lipid metabolism are essential for RNA virus replication in mosquito cells. This mechanism reveals the general law of viral RC formation, while providing an important reference for understanding the replication strategies of different mosquito-borne RNA viruses.

### 2.3 Mosquito-borne viruses disrupt host lipid metabolism and drive membrane system remodeling

The formation of mosquito-borne RNA virus RC is closely related to the reprogramming of host lipid metabolism, which is the fundamental driving force. Mosquito-borne RNA viruses typically remodel the host’s lipid metabolism pathways after infecting the host (Cheon et al., 2006). This process provides raw materials for the membrane structure of the viral RC, while supporting viral assembly and release by regulating energy metabolism and membrane dynamics (Heaton and Randall, 2011; O'Neal et al., 2020; Perera et al., 2012). DENV, ZIKV, and CHIKV can all reprogram the host lipid environment by interfering with fatty acid synthesis, cholesterol distribution, and phospholipid metabolism (O'Neal et al., 2020). Taking DENV as an example, early studies have shown that DENV infection of host cells can significantly reprogram the host lipid metabolism pathway, induce the upregulation of rate-limiting enzymes, including FAS, and recruit a variety of lipid molecules to construct the viral RC (Heaton and Randall, 2011; Perera et al., 2012). However, direct evidence for the applicability of this mechanism in mosquito-borne hosts was lacking in early studies. Subsequent researchers discovered, through techniques such as immunofluorescence and Western blotting, that DENV infection induces multiple changes in the C6/36 mosquito cell, including a decrease in lipid droplet content and an upregulation of lipid mobilization genes, which verified this conclusion in mosquitoes (Heaton and Randall, 2011; Perera et al., 2012; Farías et al., 2024). Based on this theory, Chotiwan and his team revealed that DENV affects fatty acid metabolism while reshaping multiple lipid metabolic pathways, such as cholesterol and phospholipids, thereby supporting its own replication needs (Heaton and Randall, 2011; Chotiwan et al., 2018; Vial et al., 2020; O'Neal et al., 2020; Perera et al., 2012; Xie et al., 2025). DENV interacts synergistically with lipid metabolism by regulating host autophagy pathways, energy metabolism networks, and lipid transporters, thereby establishing a highly coupled metabolic regulatory network (Bowman et al., 2016; Perera et al., 2012; Carnec et al., 2016). This supports critical life cycle processes within mosquito vectors, including replication and assembly within midgut cells, as well as midgut escape and dissemination to secondary tissues (Bowman et al., 2016; Perera et al., 2012; Carnec et al., 2016; Ocampo et al., 2013). It is important to note that even in the absence of *Wolbachia*, certain tolerant or resistant mosquito strains (e.g., *Ae. aegypti*) can inherently limit viral spread through midgut infection or barrier escape mechanisms (Ocampo et al., 2013). This confines viral replication to midgut cells, preventing systemic infection and thus blocking viral progression to salivary glands and successful transmission (Ocampo et al., 2013). ZIKV is more dependent on cholesterol metabolism, whereas CHIKV tends to regulate phospholipid and membrane structure more significantly, showing some conservation and coordination among different mosquito-borne RNA viruses (Stoyanova et al., 2023; Teppor et al., 2021; Reis et al., 2022), suggesting that this mechanism may have broader adaptive and biological implications.

Given the central role of host lipid metabolism in supporting viral replication (as described in Section 2. 3), any mechanism that disrupts this process may serve as an antiviral target. As an endosymbiotic bacterium, *Wolbachia* undermines viral life-cycle dependencies across multiple dimensions by reshaping the host’s lipid metabolic network.

## 3 *Wolbachia*’s antiviral mechanism through interference with host lipid metabolism

**Figure 6:**
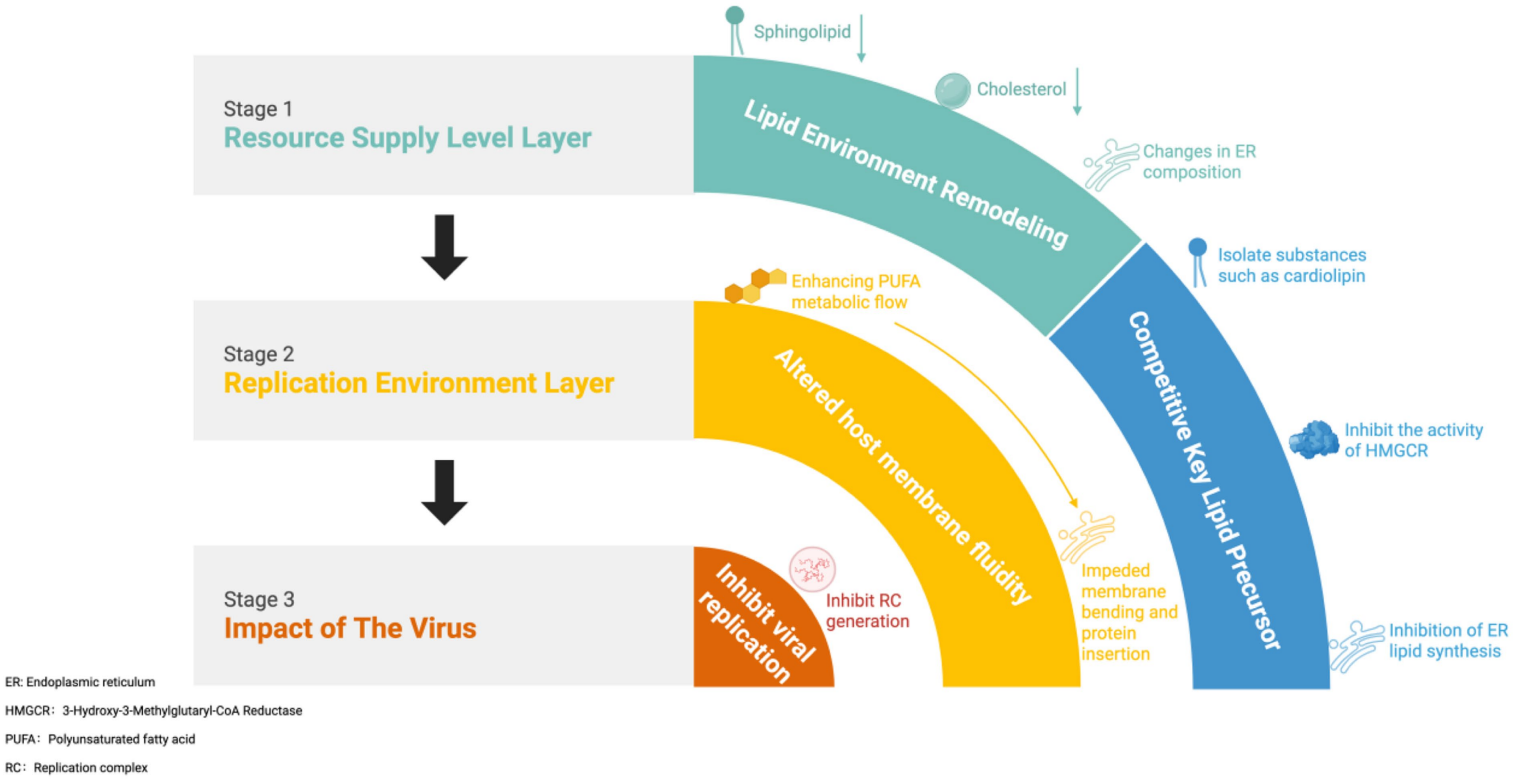
Mechanism of *Wolbachia* interference with lipid metabolism in mosquito-borne RNA viruses.

Establishing stable *Wolbachia* infection within mosquitoes profoundly reshapes the host’s lipid metabolism, thereby inhibiting the replication of RNA viruses. Viral replication relies on two core conditions: abundant lipid resources and a synthetic site with high fluidity. DENV, ZIKV, and CHIKV all require specific lipid precursors (Such as HexCer, which plays a crucial role in the replication process of CHIKV; Noble et al., 2025) to complete replication. *Wolbachia* infection initially restricts viral access to critical resources by competing for lipid precursors and altering lipid composition. It disrupts the assembly and stability of RC by altering the cholesterol-to-polyunsaturated fatty acid ratio, thereby reducing membrane fluidity and plasticity (Figure 6). Consequently, *Wolbachia* can be considered to comprehensively inhibit mosquito-borne RNA viral replication by targeting both resource supply and structural environment. It should be noted that *Wolbachia*’s regulation of lipid metabolism does not constitute an active adaptive suppression of viral replication. It likely reflects changes in the host environment driven by the bacterium’s own colonization and metabolic demands, thereby generating a secondary antiviral effect.

Given the significant differences in the ability of different *Wolbachia* strains to influence host lipid metabolism, it is necessary to distinguish between them before discussing lipid metabolism-related mechanisms. Existing studies consistently indicate that alterations in the lipid environment of mosquito hosts are markedly strain-dependent rather than a universal characteristic of this bacterial genus: different strains exhibit variations in the points of influence, the intensity of their effects, and the resulting metabolic outcomes (Loterio et al., 2024; Manokaran et al., 2020; Martins et al., 2021). The mechanisms summarized below represent only the functional pathways that have been reported and do not imply that these effects are universally present across all strains (Table 5).

**Table 5:** Representative *Wolbachia* strains, mosquito hosts, and affected viruses based on lipid-related mechanisms.

Mechanisms affecting lipid metabolism	Reported strain	Mosquito vector species	Virus affected by the influence
Lipid environment remodeling (Loterio et al., 2024; Lau et al., 2024)	*w*Mel	*Ae. aegypti*	DENV, ZIKV
	*w*AlbB		DENV, ZIKV
	*w*Pip (for comparison)		Weak antiviral activity, no significant lipid changes
Competition for key lipid precursor resources (Loterio et al., 2024; Schultz et al., 2018; Manokaran et al., 2020; Ahmad et al., 2021; Edwards et al., 2023)	*w*Mel	*Ae. aegypti*	DENV, ZIKV
	*w*AlbB		DENV, ZIKV
	*w*MelPop	*Ae. albopictus*	DENV, ZIKV
	*w*Stri		DENV, ZIKV, CHIKV, YFV
Reduced membrane fluidity (Loterio et al., 2024; Manokaran et al., 2020)	*w*Mel	*Ae. aegypti*	DENV
	*w*AlbB		DENV, ZIKV
	*w*Pip (for comparison)		Weak antiviral activity, no significant lipid changes
Regulating organelle interactions (Manokaran et al., 2020; Martins et al., 2021)(primarily mitochondria, but evidence is insufficient)	*w*Mel	*Ae. aegypti*	DENV, ZIKV
	*w*Pip		ZIKV

### 3.1 Lipid environment remodeling inhibits viral replication

Mosquito-borne RNA viruses can reshape the host membrane environment by altering the abundance of multiple lipid classes through lipidomics and cellular imaging analysis, including the upregulation of common sphingolipids such as ceramide, an increase in glycerophospholipid abundance, and cholesterol enrichment (Loterio et al., 2024; Leier et al., 2020; Perera et al., 2012; Hehner et al., 2024; Melo et al., 2018). This reshaping promotes the formation of RC and facilitates viral replication (Loterio et al., 2024; Leier et al., 2020; Hehner et al., 2024). In contrast, antiviral *Wolbachia* strains (e.g., *w*Mel, *w*AlbB) counteract these virus-favorable alterations by rearranging the host’s lipid environment (Koh et al., 2020; Molloy et al., 2016; Elliott et al., 2023). Infection can reduce sphingolipid and cholesterol levels as well as the precursors required for phospholipid synthesis, such as PI and phosphatidylcholine (PC), and alter the lipid composition of the ER, thereby disrupting the establishment of viral replication sites (Loterio et al., 2024; Molloy et al., 2016; Elliott et al., 2023); *w*Mel and *w*AlbB can induce lipid droplet formation and associate with the endomembrane system, restricting viral access to these resources by isolating key membrane lipids (Loterio et al., 2024; Geoghegan et al., 2017) (Table 6). Therefore, the core mechanism by which *Wolbachia* interferes with viral replication lies in remodeling the host’s lipid environment from a cellular state conducive to viral replication to one that is restrictive for the virus, a finding repeatedly confirmed by multi-omics and biochemical studies (Loterio et al., 2024; Koh et al., 2020; Geoghegan et al., 2017; Molloy et al., 2016; Elliott et al., 2023). This suggests that future research should aim to pinpoint the lipid categories most causally involved in antiviral effects by comparing lipid profiles across different *Wolbachia* strains or host species.

**Table 6:** Lipid environment reconfiguration.

Mechanism	Virus regulation	*Wolbachia* interference
Lipid composition remodeling (Loterio et al., 2024; Koh et al., 2020; Leier et al., 2020; Perera et al., 2012; Hehner et al., 2024; Melo et al., 2018; Molloy et al., 2016; Elliott et al., 2023)	Upregulation of common sphingolipids such as ceramide; Increased glycerophospholipid abundance; Enrichment cholesterol.	Antiviral *Wolbachia* strains reduces sphingolipid and cholesterol levels; Diminishes precursors such as PI and PC; Alters ER lipid composition and disrupts RC establishment.
Lipid Regulation (Loterio et al., 2024; Geoghegan et al., 2017)	Virus-mediated mobilization of lipid droplets provides membrane lipid resources.	*w*Mel and *w*AlbB induces lipid droplet formation and associates with the endomembrane system; Sequestering key lipids.

PI, phosphatidylinositol; PC, phosphatidylcholine; ER, endoplasmic reticulum; RC, replication complex.

### 3.2 Competition for key lipid precursor resources

A metabolomics analysis validated and confirmed that *w*Mel and *w*MelPop strains may also suppress viral replication by competing with viruses for the host’s limited metabolic and lipid resources, as shown through metabolomics analysis (Geoghegan et al., 2017; Manokaran et al., 2020; Molloy et al., 2016). Mosquito-borne RNA viruses require substantial amounts of specific substrates such as sphingomyelin, cardiolipin, acyl-carnitines, and cholesterol during early infection to construct the membrane structure and energy supply system of the RC (Geoghegan et al., 2017; Manokaran et al., 2020; Molloy et al., 2016). Following specific *Wolbachia* strains (e.g., *w*Stri, Schultz et al., 2018; *w*MelPop, Edwards et al., 2023) infection, these metabolic pathway products are sequestered, leading to the depletion of cholesterol and its synthetic precursors (Schultz et al., 2018; Edwards et al., 2023). This interferes with the activity of HMG-CoA reductase (3-Hydroxy-3-Methylglutaryl-CoA Reductase, HMGCR), the rate-limiting enzyme in the mevalonic acid pathway (MVA), and alters the synthetic flux of sphingomyelin and phospholipids, thereby reducing the availability of key membrane lipids for the virus (Edwards et al., 2023). Metabolic modeling analysis predicts that high-density colonizing *w*Mel and *w*MelPop gain competitive advantage in the race for limited host metabolic resources, thereby reshaping host metabolic flux and restricting key metabolic pathways essential for viral replication (Jiménez et al., 2021). These include limiting the supply of substances such as mitochondrial membrane-associated cardiolipin and inhibiting the resynthesis of endoplasmic reticulum lipids (Jiménez et al., 2021). This conclusion is consistent with the findings of metabolomics analysis. Subsequent functional validation experiments confirmed that supplemental acylcarnitine partially reversed the suppression of viral replication by high-density *w*Mel (Manokaran et al., 2020) (Table 7). This finding complements the theoretical framework of the “resource competition (Jiménez et al., 2021)” model–*Wolbachia* does not simply suppress metabolism globally, but instead directly competes with viruses for specific lipid precursors in both time and space (Jiménez et al., 2021; Koh et al., 2020; Geoghegan et al., 2017). This perspective warrants investigation, and prospective studies could employ dynamic metabolic tracing to elucidate the temporal sequence of their competition for the same substrates.

**Table 7:** Competition for key lipid precursor resources.

Mechanism	Substrates required by virus	*Wolbachia* interference
Lipid precursor competition (Geoghegan et al., 2017; Manokaran et al., 2020; Molloy et al., 2016)	Sphingomyelin; Cardiolipin; Acyl-carnitines; Cholesterol.	Alter the metabolic flux of sphingomyelin; Disrupt the activity of HMG-CoA reductase in the mevalonate pathway; Seize metabolic pathway products, consuming cholesterol and its precursors.
Metabolic flow constraints (Jiménez et al., 2021)	Membrane-associated cardiolipin Fatty acid; PI; PC.	Limit the supply of substances such as mitochondrial; Reduce membrane-associated cardiolipin and inhibit the resynthesis of endoplasmic reticulum lipids.
Functional validation (Manokaran et al., 2020)	—	Exogenous supplementation of acyl-carnitines partially reverses the inhibitory effects of *w*Mel.

HMG-CoA, 3-hydroxy-3-methylglutaryl-coenzyme A; PI, phosphatidylinositol; PC, phosphatidylcholine.

### 3.3 Reduced membrane fluidity restricts replication complex assembly

Host cell membrane fluidity is associated with multiple factors, including the structural composition of polyunsaturated fatty acid (PUFA) chains, cholesterol content, and lipid-protein interactions (Lund et al., 1999; Khodadadi et al., 2025; Muriana et al., 1992; Torres et al., 2020). Membrane fluidity reflects the functional properties of the lipid environment and plays a crucial role in regulating host-virus interactions. Mosquito-borne RNA viruses enhance membrane plasticity by increasing unsaturated fatty acids and cholesterol, thereby facilitating the embedding and bending of RC (Loterio et al., 2024; van den Elsen et al., 2021). For instance, after DENV infects mosquito hosts, it actively activates and hijacks the host cell’s phospholipid remodeling cycle, inducing increased activity of phospholipase A2 (PLA2) to generate large amounts of hemolytic phospholipids and free fatty acids (Vial et al., 2020). Simultaneously, it utilizes LPCAT activity to elevate unsaturated fatty acids, thereby enhancing membrane fluidity and supporting RC formation (Vial et al., 2020). In contrast, upon specific *Wolbachia* strains infection, the host undergoes a reshaping of its membrane lipid composition, with reduced levels of cholesterol and its precursors (Loterio et al., 2024; Molloy et al., 2016). The metabolic flux of PUFAs is also enhanced, diminishing ER membrane fluidity and impeding the membrane bending and protein insertion processes required for viral RC (Loterio et al., 2024; Molloy et al., 2016). Different experimental teams reached consistent conclusions from lipid profile analysis and enzyme activity assays, indicating that the reduction in membrane fluidity represents a core point in *Wolbachia*’s antiviral mechanism (Loterio et al., 2024; Vial et al., 2020; Molloy et al., 2016; van den Elsen et al., 2021). However, this inference currently remains largely based on observational correlations. Functional validation in this regard remains limited, with existing studies only demonstrating that supplementation with acyl-carnitines reverses *w*Mel’s suppression of viral replication (Manokaran et al., 2020). No studies have yet attempted to restore membrane fluidity by exogenously adding cholesterol or specific unsaturated fatty acids to *Wolbachia*-infected cells, nor have they investigated whether such interventions could reverse the bacteriophages’ inhibitory effect on viral replication (Table 8). There remains a lack of critical evidence to distinguish whether the decline in membrane fluidity is the driving factor behind *Wolbachia*’s suppression of viral replication or merely a concomitant phenomenon of replication inhibition.

**Table 8:** Reduced membrane fluidity restricts replication complex assembly.

Mechanism	Virus regulation	*Wolbachia* interference
Membrane fluidity (Vial et al., 2020)	Elevates levels of unsaturated fatty acids and cholesterol; Activates PLA2 to generate hemolytic phospholipids and free fatty acids; LPCAT increases PUFA, enhancing membrane plasticity.	Reduce cholesterol and its precursor substances; Enhance the metabolic flow of polyunsaturated fatty acids; Decrease ER membrane fluidity.
Functional validation testing (Manokaran et al., 2020) (Insufficient)	—	Acyl-carnitine supplementation partially reversed the inhibitory effects of *w*Mel but lacks the functional effects of cholesterol and PUFA supplementation.

PLA₂, phospholipase A₂; LPCAT, lysophosphatidylcholine acyltransferase; PUFA, polyunsaturated fatty acids; ER, endoplasmic reticulum.

### 3.4 Other mechanisms

Specific *Wolbachia* strains may also indirectly influence lipid distribution by regulating interactions between organelle. The dynamic interaction between mitochondria and lipid droplets plays a crucial role in cellular energy metabolism and lipid homeostasis, providing a metabolic basis for the establishment of RC (Fan and Tan, 2024). However, evidence for this mechanism in specific *Wolbachia* strains infection remains relatively insufficient. Proteomics combined with functional pathway analysis revealed that *w*Pip infection increases mitochondrial reactive oxygen species levels and forms barrier-like structures by binding to lipid droplets (Martins et al., 2021). This approach restricts viral resource acquisition while simultaneously suppressing viral replication efficiency through oxidative stress (Martins et al., 2021; Yin et al., 2020). The aforementioned experiments demonstrate that *w*Mel infection depletes acyl-carnitines, thereby blocking fatty acid transport into mitochondria, which in turn impairs *β*-oxidation and reduces ATP synthesis, causes abnormal lipid droplet storage, and ultimately maintains viral replication at low levels due to insufficient energy and substrates (Manokaran et al., 2020). These findings collectively point to a relatively consistent understanding that the interaction between lipid droplet and mitochondria represents a critical node in *Wolbachia*’s regulation of metabolism, where disruption impairs both lipid mobilization and energy supply (Martins et al., 2021; Fan and Tan, 2024; Yin et al., 2020). However, the core mechanisms they emphasize are not entirely consistent, with Martins’ research placing greater emphasis on the immediate defensive role of oxidative stress (Martins et al., 2021), while Manokaran’s findings underscore the long-term inhibitory effects resulting from energy deprivation (Manokaran et al., 2020). A reasonable assumption is that specific *Wolbachia* strains may simultaneously activate multiple pathways, with its dominant mechanism potentially varying based on host age, infection stage, or environmental temperature, revealing the diversity of this mechanism. However, most evidence for this mechanism relies on omics analyses or speculative models (Cheng and Simmonds, 2025; Thind et al., 2024), and direct super-resolution or dynamic imaging evidence remains lacking. Although these speculations draw partially on findings from mammalian or other model organisms, they have already suggested the critical role of the interaction between lipid droplets and mitochondria in fatty acid mobilization, *β*-oxidation for energy production, and redox balance (Kien et al., 2022). The above findings suggest that *Wolbachia*’s regulation of lipid metabolism contributes to its antiviral effects through the coordinated action of multiple pathways. Moving forward, researchers need to elucidate the temporal and spatial causal relationships among these mechanisms, while also identifying the dominant mechanism under specific conditions.

## 4 Summary and outlooks

Recent studies have demonstrated that *Wolbachia* disrupts host lipid metabolism through multi-level, multi-pathway mechanisms, thereby effectively suppressing the replication of RNA virus within mosquitoes. Its core functions include rearranging the host lipid environment, competing with viruses for key metabolic resources, regulating interaction between mitochondrial and lipid droplets, and altering membrane fluidity. These mechanisms interact to form a highly coupled metabolic regulatory network, imposing multiple constraints on the assembly and function of viral RC, thereby achieving significant suppression of viral transmission. Notably, different *Wolbachia* strains exhibit variations in lipid regulation capacity and antiviral spectrum, suggesting that their antiviral effects depend on both host-virus interaction characteristics and the regulation of the microorganism’s own properties.

Based on this theoretical foundation, extensive clinical evidence indicates that *Wolbachia* can markedly suppress the replication and transmission of multiple mosquito-borne RNA viruses, including DENV, ZIKV, and CHIKV (Edenborough et al., 2024). Both *w*Mel and *w*AlbB strains effectively reduce viral infection rates, replication levels, and transmission potential within *Ae. aegypti* in both laboratory and natural populations (Lim et al., 2024b; Ahmad et al., 2021). Notably, *w*AlbB maintains high density and stable maternal transmission even under high-temperature conditions, making it suitable for deployment in tropical regions (Mancini et al., 2021). Different strains exhibit specific suppression capabilities against distinct mosquito-borne viruses. Regions should adapt their approaches based on local conditions. Drawing from past field trials, cultivating mosquito strains co-infected with multiple strains can effectively eliminate mosquito vectors while simultaneously suppressing the transmission of mosquito-borne viruses.

It should be noted that while existing research generally supports the inhibitory effect of *Wolbachia* on various mosquito-borne viruses, this effect is not consistently observed across all viruses and experimental systems (Lim et al., 2024b; Ahmad et al., 2021; Edenborough et al., 2024). Some studies report that under specific host-virus combinations, the impact of *Wolbachia* on certain mosquito-borne viruses is limited or insignificant, and in isolated cases, it has even been associated with elevated viral infection levels (Dodson et al., 2023; Lefteri et al., 2024; Johnson, 2015; Mousson et al., 2012). Compared to the inhibitory effects of most positive-strand RNA viruses, the impact patterns of *w*AlbB and *w*Mel on small RNA viruses are distinctly different: in *Ae. aegypti* mosquitoes, mosquitoes harboring *w*AlbB or *w*Mel exhibit enhanced susceptibility to Sinudis virus (SINV) infection, while demonstrating a strain-independent neutralizing effect against ONNV (Dodson et al., 2023). For Mayaro virus (MAYV), *w*AlbB had no significant impact on viral infection, whereas *w*Mel demonstrated marked suppression (Dodson et al., 2023). Conversely, in *Wolbachia*-infected *Ae. aegypti* mosquitoes (carrying *w*Mel, *w*AlbB, or *w*Au strains), the suppression of the negative-strand RNA virus Bunyamweira virus was not significant, contradicting the viral suppression observed *in vitro* (Lefteri et al., 2024). In naturally infected *w*AlbA/*w*AlbB *Ae. albopictus*, the overall viral load of DENV and CHIKV was not significantly lower than in mosquito populations lacking *Wolbachia* (Johnson, 2015; Mousson et al., 2012). These findings suggest that *Wolbachia*-mediated antiviral effects may be context-dependent, with their specific manifestations regulated by multiple factors including virus type, *Wolbachia* strain, and host physiological state. Preventing the reversal of viral suppression by *Wolbachia* strains is crucial.

The application of *Wolbachia* in controlling mosquito-borne transmission has achieved remarkable success, but the mechanisms underlying the interactions among the virus, mosquito, and investigation of *Wolbachia* remains necessary. The following directions outline research priorities. On one hand, it is necessary to utilize multi-omics-based metabolic modeling and functional validation approaches to clarify the causal regulation of *Wolbachia* on key nodes of lipid metabolism and its universality and specificity in replicating different RNA viruses. On the other hand, attention should be paid to the mechanisms underlying the roles of mitochondrial-lipid droplet interactions, changes in membrane fluidity, and the spatiotemporal distribution of metabolic resources in antiviral processes, as well as exploring their dynamic regulatory patterns within the host cell microenvironment. The multi-strain co-infection strategy also demonstrates significant application potential in the prevention and control of mosquito-borne diseases. Compared to current field releases primarily relying on single strains (such as *w*Mel or *w*AlbB), the development of customized multi-strain co-infected mosquitoes combined with low-dose radiation sterilization strategies for female mosquitoes holds promise. This approach may enhance the broad-spectrum efficacy and long-term stability of antiviral effects while maintaining population controllability, offering a new technical direction for next-generation Wolbachia control strategies.
